# Breaking bad news: Awareness and practice among Sudanese doctors

**DOI:** 10.3934/publichealth.2020058

**Published:** 2020-09-24

**Authors:** Mumen Abdalazim Dafallah, Esraa Ahmed Ragab, Mahmoud Hussien Salih, Wail Nuri Osman, Roaa Omer Mohammed, Mugtaba Osman, Mohamed H. Taha, Mohamed H. Ahmed

**Affiliations:** 1 Faculty of Medicine, University of Gezira, Sudan; 2 Armed Forces Centre for Psychiatric Care, Taif, Saudi Arabia; 3 College of Medicine and Medical Education Center, University of Sharjah, Sharjah, United Arab Emirates; 4 Department of Medicine and HIV Metabolic Clinic, Milton Keynes University Hospital NHS Foundation Trust, Eaglestone, Milton Keynes, Buckinghamshire, UK

**Keywords:** breaking bad news, Sudan

## Abstract

**Background:**

Breaking bad news is an important task for doctors in different specialties. The aim of the study was to assess adherence of Sudanese doctors to the SPIKES protocol in breaking bad news.

**Methods:**

A descriptive cross-sectional study recruited 192 doctors, at Wad Medani teaching hospital, Sudan. A questionnaire-based on SPIKES protocol was distributed among 10 departments in our hospital. Data were analyzed using SPSS and Microsoft excel.

**Results:**

There were (n = 101, 52.6%) females and (n = 91, 47.4%) males among the participants. 95.3% have been involved in breaking bad news, but only 56.3 received education and training about this issue. 43% admitted bad experience in breaking bad news, while 65.6% mentioned that bad news should be delivered directly to patients. The majority (>90%) agreed training is needed in the area of breaking bad news. Usual adherence to the SPIKES protocol was reported in a range of 35–79%, sometimes adherence was reported in a range of 20–44% while never adherence was reported in a range of zero–13.5%. Consultants, registrars, obstetrician and gynecologists and surgeons achieved high scores in breaking bad news. Training is an important factor in achieving high score in SPIKES protocol. The unadjusted effect of background factors on SPIKES score, showed that only training has significant impact on protocol adherence (P = 0.034, unadjusted; and P = 0.038 adjusted).

**Conclusion:**

Large number of Sudanese doctors will try to adhere to SPIKES protocol. Training is an important factor in the success of breaking bad news.

## 1. Introduction

One of the common and most important tasks for practicing clinician is breaking bad news to his patients. Buckman defined bad news as “any news that drastically and negatively alters the patient's view of her or his future” [1]. It is possible to include in the definition of bad news other conditions in which there is “a feeling of no hope” or “a message is given which conveys to an individual fewer choice” [2]. Therefore, even experienced doctor may find certain occasions of breaking bad news required considerable preparations [3]. This can be due in part that some doctors may worry about being blamed, the reactions associated with breaking bad news, and fear of not knowing what will happen especially among young doctors [4]. Importantly, breaking bad news if not well delivered may have an impact on the clinician and patients and relatives. For instance, physicians may become emotionally disengaged from their patients, and its possible this may have an impact on patients or relatives relationship with their doctors [5],[6]. Breaking badnews is widely practiced in hospital especially with debilitating illnesses, such as stroke, myocardial infarction, dementia, multiple sclerosis and Parkinson's disease [7],[8]. Several protocols have been proposed to communicating bad news and tested in the literature. However, there are a few established recommendations for the delivery of bad news in the United States [9], Germany [10] and the United Kingdom [11]. These protocols are primarily based more on expert opinion, but less on empirical evidence.

The most popular guideline, the SPIKES protocol [12], recommends six-steps for breaking bad news, with a special application for cancer patients [13]. It was evaluated for structuring the delivery of bad news in the United States [14], Germany and other countries [10]. It is used as a guide for this sensitive practice and for communication skills training in this context [15]. The first step is S or setting up phase that points to the preparation of the medical environment, which should preferably be a private, reserved, and pleasant site. The second step is P or perception; it is an opportunity to find what the patient knows about his or her illness through open questions. The third stage is I or invitation is an opportunity to analyze the patient's willingness rate to resolve his doubts about his disease. The fourth stage is the K or knowledge that everything in relation to the diagnosis must be revealed. The fifth stage is the E or emotion, which is the time to express empathy, recognize the patient's emotions, and provide support. The last step is the S or phase of strategy and summary that is the moment to propose treatment and prognosis of the disease, as well as sum up everything that has been said[16],[17].

Other protocols are also available like patient- and family-centered approach [18] and an emotion-centered approach [19]. For example, Rabow and McPhee also proposed a model for breaking bad news called ABCDE: A, advance preparation; B, build a therapeutic environment/relationship; C, communicate well; D, deal with patient and family reactions; and E, encourage and validate emotions [6]. There is limited data endorsing the use of the SPIKES protocol in Sudan. However. within the last decade, Sudanese doctors become familiar with SPIKES protocol as most of the international postgraduate medical examinations (mainly from UK) were held in the capital Khartoum. These examinations will include one station at least about breaking bad news.However, in the daily practice of medical doctors, it's very likely to see wide variation in adherence to SPIKES protocol and this can be due to cultural, religious and customs in different parts of Sudan. In this study, we assessed the use of SPIKES protocol in breaking bad news among Sudanese doctors working at Wad Medani teaching hospitals, Sudan.

## 2. Methodology

This is a cross-sectional study conducted from August to December 2019, at Wad Medani teaching hospitals, Gezira state, central Sudan. A self-administered questionnaire was designed to elicit information on doctor's knowledge and practices about breaking bad news to patients and their relatives. The questionnaire was distributed among 10 departments (medicine, surgery, pediatric, pediatric surgery, urology, nephrology, ENT, orthopedic, oncology, obstetrics and Gynecology departments). Medical officers, residence registrars and consultants working in Wad Madani teaching hospitals and agreed to fill the questionnaire were included in this study. The following were excluded: house officers in those hospitals departments, doctors who did not have direct contact with patients (radiologists and pathologists) and doctors who refuse to participate or not available at the time of the study. Participants received questinnare with covering letter with details of projects, right of responsendents and confidentiality of the data.

The questionnaire consisted of three sections. The first section was personal data including age, gender, clinical position and specialty. The second section was related to their practice about breaking bad news which composed of sex items. Each item was measured on a 3-point Likert scale (usually, sometimes and never). The items were based on the SPIKES model of breaking bad news. The third section asks the doctors about their past experiences, opinions and the need for a training program in breaking bad news which composed of 11 items. Acceptance of hospitals administrations on approval was also taken. The data obtained were secured and kept confidential and were used only for research purposes.

### 2.1. Method of determination sample size

192 doctors were enrolled in this study, stratified by random sampling. As we intend to utilize a mixed linear model, we estimated the sample size assuming a small effect size (Cohen's d = 0.15), *with ten different clusters (representing the specialties of participating doctors), and power of 0.8*, significance of 5%. The minimum required sample size is (n = 159) doctors. (Cohen J. 1988. Statistical power analysis for the behavioral sciences (2nd ed.).

### 2.2. Statistical analysis

The data were analyzed using SPSS (Statistical Package for Social Sciences) version 23 and Microsoft excel. Descriptive statistics were applied to describe the pattern of the data In terms of following SPIKES protocols, we summed up the score for the six SPIKES responses. This gives a potential total of 12 for perfect adherence to the protocol. Logistic regression analysis was used to determine factors that have an impact on breaking bad news.

### 2.3. Ethical approval

Was obtained from the Research Ethical Committee of the University of Gezira—Faculty of Medicine on September 11th, 2019.

## 3. Results

### 3.1. Sociodemographic features of participants

The data included responses from (n = 192) participants. There were (n = 101, 52.6%) females and (n = 91, 47.4%) males among the participants. The most frequent age of participants was less than 30 (n = 113, 58.9%), followed by those between 30 and 40 (n = 51, 26.6%) then over 40 (n = 28, 14.6%). Most of the participants (n = 96, 50%) were trainee registrar, followed by (n = 56, 29.2%) medical officers, and (n = 40, 20.8%) who were practicing consultants (table 1).Physicians were the most participating doctors (n = 39, 20.3%), followed by obstetricians (n = 32, 16.7%), general surgeons (n = 25, 13.0%), and orthopedic surgeons (n = 23, 12.0%). Pediatricians constituted (n = 22, 11.5%) of participants, with oncologists, urologists, pediatric surgeons were (n = 11, 5.7%). Nephrologists were (n = 10, 5.2%) and ENT surgeons were (n = 8, 4.2%) (Table 1).

**Table 1.** Demographic characteristic of participants.

	Frequency	%
Age		
Less than 30 years	113	58.9
From 31 to 40 years	51	26.6
More than 41 years	28	14.5
Gender
Male	91	47.4
Female	101	52.6
Clinical position		
Consultants	40	20.8
Registrars	96	50
Medical officers	56	29.2
Specialty		
Medicine	39	20.3
General surgery	25	13
Obstetrics and gynecology	32	16.7
Pediatric	22	11.5
Pediatric surgery	11	5.7
Orthopedic	23	12
Urology	11	5.7
Nephrology	10	5.2
Oncology	11	5.2
ENT	8	4.2

### 3.2. Knowledge, training and experience

95.3% have been involved in breaking bad news, but only 56.3 received education and training about this issue. 43% admitted bad experience in breaking bad news, while 65.6% mentioned that bad news should be delivered directly to patients. The majority (>90%) agreed training is needed (Table 2).

**Table 2.** Showing answers to different questions (knowledge, training and experience) about breaking bad news.

Item	Yes (%)	No (%)
1. Have you ever received any education/training for breaking bad news?	108 (56.3%)	84 (43.8%)
2. Have you ever broken bad news to patients or patients' family	183 (95.3%)	9 (4.7%)
3. Did you have any bad experiences due to improperly breaking bad news?	84 (43.8%)	108 (56.3%)
4. Do you prefer to talk with a patient or the family members when you break bad news?	Patient (n = 81, 42.2%)	Family & patient (n = 111, 57.8%)
5. Do you believe that bad news should be delivered directly to the patient?	126 (65.6%)	66 (34.4%)
6. Do you feel training is needed for adequate skill development in breaking bad news	182 (94.8%)	10 (5.2%)
7. Are you willing to attend training regarding breaking bad news in the future?	183 (95.3%)	9 (4.7%)

### 3.3. Adherence to SPIKES protocol by specialty, rank and training

**Figure 1.**
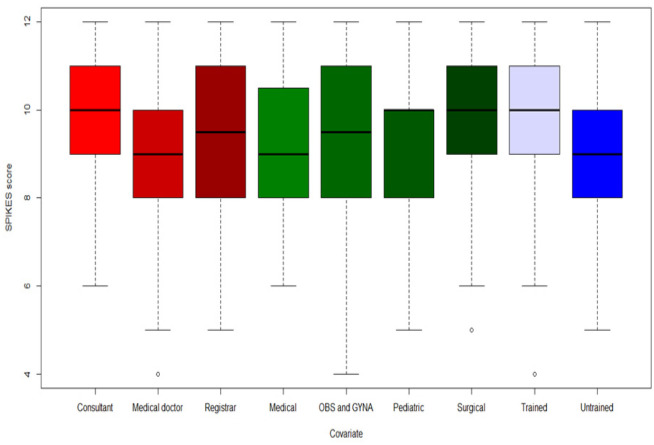
showed the SPIKES protocol with potential of 12 for perfect adherence to the protocol. Consultants, registrars, obstetrician and gynecologists and surgeons achieved high scores in breaking bad news. Training is an important factor in achieving high score in SPIKES protocol.

Adherence to the SPIKES protocol can be seen in Table 3. Usual adherence to protocol was reported in a range of 35–79%, sometimes adherence was reported in a range of 20–44% while never adherence was reported in a range of zero–13.5% (Table 3). In terms of following SPIKES protocols, we summed up the score for the six SPIKES responses. This gives a potential total of 12 for perfect adherence to the protocol. The mean score was 9.3 (SD = 1.78). The responses ranged between 4 and 12, and the median was 10. The maximum of 12 was achieved by (n = 23, 12%) doctors (Table 4). Figure 1 showed the SPIKES protocol with potential of 12 for perfect adherence to the protocol. Consultants, registrars, obstetrician and gynecologists and surgeons achieved high scores in breaking bad news. Training is an important factor in achieving high score in SPIKES protocol. The unadjusted effect of background factors on SPIKES score, showed that only training has significant impact on protocol adherence (P = 0.0341, unadjusted; and P = 0.038 adjusted) (Table 5).

**Table 3.** Participants' Adherence to SPIKES protocol.

Item	Never (%)	Sometimes (%)	Usually (%)
1 S. Do you set up (plan) the interview for the patient to feel comfortable and keep privacy?	9 (4.7%)	85 (44.3%)	98 (51%)
2 P. Do you assess the patient's perception (what he already knows) about the condition?	6 (3.1%)	65 (33.9%)	121 (63%)
3 I. Do you obtain the patient's invitation (ask him what they want to know)?	26 (13.5)	94 (49%)	72 (37.5%)
4 K. Do you give knowledge and information to the patient about its condition?	0	40 (20.8%)	152 (79.2%)
5 E. Do you assess the patient's emotions with emphatic responses?	7 (3.6%)	77 (40.1%)	108 (56.3%)
6 S. Do you explain future strategy including treatment options and prognosis?	3 (1.6%)	46 (24%)	143 (74.5%)

**Table 4.** In terms of following SPIKES protocols, we summed up the score for the six SPIKES responses. This gives a potential total of 12 for perfect adherence to the protocol. The mean score was 9.3 (SD = 1.78). The responses ranged between 4 and 12, and the median was 10. The maximum of 12 was achieved by (n = 23, 12%) doctors.

SPIKES score	4	5	6	7	8	9	10	11	12
Count	1	4	12	10	27	40	48	27	23
Percentage	0.5%	2.1%	6.3%	5.2%	14.1%	20.8%	25%	14.1%	12%

**Table 5.** The unadjusted effect of background factors on SPIKES score, only training has significant impact on protocol adherence (P = 0.03416, unadjusted; and P = 0.038 adjusted).

	Unadjusted analysis			Adjusted mixed linear analysis			
Factor	Effect (mean)	t test/F value	P value	Estimate	Standard Error	t value	P value
Training							
Yes	9.593	2.136	0.034	0.549	0.264	2.076	0.038
No	9.036			Reference			
Discipline							
Medical	9.300	0.093	0.964				
Surgical	9.423			Random effect	NA	NA	NA
Obstetrics	9.250						
Paediatrics	9.364						
Rank			0.494				
Consultant	9.575	0.708		Reference	NA	NA	NA
Registrar	9.375			0.002	0.548	0.004	0.997
Medical officer	9.143			−0.165	0.633	−0.261	0.794
Gender			0.887				
Female	9.366	0.143		Reference	NA	NA	NA
Male	9.330			−0.069	0.281	−0.245	0.806
Age Category							
Less than 30	9.212	0.814	0.445	−0.221	0.353	−0.627	0.502
30–40	9.529			Reference	NA	NA	NA
Over 40	9.571			0.141	0.573	0.246	0.806

## 4. Discussion

Communication skills and in particular breaking bad news is regarded as one of the important skills for clinician with regular contact with patients [20],[21]. The aim of this study was to explore Sudanese doctor's awareness and evaluate practice in the relation of breaking bad news to patients and their relatives. Adherence to SPIKES protocol was found to be 80%, 84.3% in Korea and Brazil respectively [22],[23]. We showed that majority of doctor usually or sometimes adhere to the SPIKES protocol, while never adherence was reported in a range of zero–13.5%. Bad experience with breaking bad news is inevitable outcomes in certain occasions. In our study, we showed that 43.8% have such experience, this percentage almost similar to studies in Nigeria and Korea [22],[24].

This was attributed to no training or education, which is common problems across the different countries like USA and Nigeria [24],[25] It worth mentioning, education per se will not improve communication in breaking bad news unless its combined with training [26]. Training will give opportunity to overcome stress associated with breaking bad news and help clinicians to develop confidence [27],[28]. The variability in the adherence to the six steps of the SPIKES in our study, was also shown in different studies [10],[11],[23],[24]. Interestingly, registrars showed equivalent level of consultants in adherence to the SPIKES protocol. This can be attributed, doctors in training are more likely to stick to guidelines in preparation for final local medical postgraduate exam in Sudan or the international medical postgraduate examinations, and at one stage may score better than their consultants [29]. Logistic regression analysis showed that training and education is an important factor in achieving excellent adherence to SPIKES protocol. Training and education is important for all clinician irrespective of their specialists. It worth mentioning, in pediatric its important to be trained in breaking bad news to families [27],[28],[30].

Culture, beliefs, patient level of education, traditions and religious can have an impact on delivering bad news. For instance, in Brazil and Sudan, families are likely to be heavily involved in the patient choice and decision and this may explain why 34.6% of participants in this study believe that bad news should be shared with family [20],[23],[31]. While 65.6 of the participants believed that bad news should be delivered directly to the patient and this can be attributed for growing trend of respecting patient right and confidentiality as stated by Sudan medical council. In addition, the era of internet and globalization contributed significantly in patient's education. Sudan, like other countries in the Middle East and the far East, where breaking bad news is delivered to the family rather than directly given to the patients. Muneer et al., showed that about 50% of Sudanese patients do not like to know their diagnosis according to the opinion of their doctors, and 20% of doctors would conceal the diagnosis from a patient upon the request of the relatives. Importantly, under one-quarter of doctors followed a standard protocol. They have also showed most of the doctors agreed that patients have the right to know everything about their diagnosis [32]. Elsiddek et al. showed only 25% of Sudanese patients with gastrointestinal malignancies were told about their correct diagnosis, the rest of patients were told they have mass or lump [33]. Salem and Salem mentioned that the trend in most countries in Middle East in breaking bad news will change with time and will be patient centred approach [34]. They have also developed a model of breaking bad news in Muslim countries based on mnemonic IGAD ( I for interview, G or gather information or background, A for assess family and religion views, A for achieve rapport and D for disclosure of information to the patient or the family based on physician use of IGAD). We recommend a new approach based on patient-family centred approach in breaking news in stages. Sudanese society and family structure is still based on extended family style and majority of families are living in extended and interconnected families and houses. The COVID-19 pandemic brought high level of unity and solidarity among Sudanese families and communities. Importantly, there is change and shift in attitude and acceptance of the terminal illness. Smart phones, technologies and internet contributed in increasing awareness and importantly how to seek medical help. Therefore, the new approach based on patient-family centred approach in stages may have the opportunity to fit within the Sudanese culture. This approach is based in giving the same information to the patient and family gradually and in stages. For instances, during clinical examination, patient and family will be told about the differential diagnosis and in the second interview both family and patient will be told the diagnosis.This may sound as simple approach, but one benefit we as physicians we do not appreciate, is fact that the extended families and interconnected communities during COVID-19 pandemic provided extensive support not only for patients but also for families. In other words, extended families and interconnected communities may provide similar support to the palliative support provided in Western societies. This may also bring degree of satisfaction for patient and family as terminal illness and end of life bring reconciliation and forgiveness among extended families and interconnected communities especially in Sudan. Indeed, further research will be needed to assess if this model of breaking bad news can be useful in Sudan.

This study is not without limitations. The cross-sectional design of the study may not allow for the temporal relationship. The participants were recruited from Wad Madani Teaching Hospital, therefore its not possible to generalize the result for the whole Sudan. Another important limitations, The SPIKES protocol is a mnemonic to remind clinicians of important steps in the delivery of bad news, but each bad news encounter is different, and not every step may be relevant in every clinical situation. Self-reported recall of whether each of these steps is followed never, sometimes, or always in practice is subjective and may be biased (clinicians may perceive that they do this more often or better than they do in reality). Despite these limitations we believe this study is novel, and it showed that training is an import factor in breaking bad news.

## 5. Conclusion and recommendation

Breaking bad news is a fundamental doctor's skill. Considerable weight in undergraduate medical curricula should be considered for building communication skills. Moreover, frequent Continuing Professional development (CPD) for doctors is required to develop these skills to be able and confident in breaking bad news for better health care delivery.
